# Current status and influencing factors of self-management in knee joint discomfort among middle-aged and elderly people: a cross-sectional study

**DOI:** 10.1186/s12877-023-04334-x

**Published:** 2023-09-29

**Authors:** Yabin Guo, Peipei Zhao, Biyun Zeng, Manman Su, Yang Zhou, Xiaotong Liu, Yang Zhou

**Affiliations:** 1 Teaching and Research Section of Clinical Nursing, Xiangya Hospital of Central South University, Changsha, China; 2 Xiang Ya Nursing School, Central South University, Changsha, China

**Keywords:** Knee joint, Middle-aged and Elderly people, Self-management, Unpleasant symptoms

## Abstract

**Background:**

This study aims to identify the current status and factors influencing self-management of knee discomfort in middle-aged and elderly people in China.

**Methods:**

A stratified multistage cluster sampling method was used to select participants from communities in China from January 15 to May 31, 2020. A cross-sectional survey was conducted using the general information questionnaire and the Knee Joint Discomfort Self-management Scale. Univariate analysis and a generalized linear model were used to analyze the factors influencing self-management.

**Results:**

The prevalence of knee discomfort was 77%. Moderate to severe discomfort accounted for 30.5%. The average item score of self-management in 9640 participants was 1.98 ± 0.76. The highest and lowest levels were: ‘daily life management’ and ‘information management’. Gender, ethnicity, education level, economic source, chronic disease, knee pain in the past month, and the degree of self-reported knee discomfort were significant predictors of self-management.

**Conclusion:**

The self-management of knee discomfort in middle-aged and elderly people is poor, and the degree of discomfort is a significant predictor. Healthcare providers should consider socioeconomic demographic and clinical characteristics to help these individuals improve their self-management skills. Attention should also be given to improving their ability to access health information and making them aware of disease risks.

**Supplementary Information:**

The online version contains supplementary material available at 10.1186/s12877-023-04334-x.

## Background

Osteoarthritis(OA) is a degenerative chronic bone and joint disease that seriously endangers the health of middle-aged and elderly people and is the most common joint disease in the United States. Approximately 32.5 million adults are diagnosed with degenerative arthritis each year [1]. Knee osteoarthritis(KOA) is currently the most common knee disease in middle-aged and elderly people [2]. In the most severe cases, joint replacement is needed, placing a physical and financial burden on the patient [3]. It has reported that more than 80% of patients lack awareness of KOA [4], which leads to its prevention and treatment being neglected. In the guidelines for KOA [5], early prevention is the main focus. Therefore, timely and effective management of knee joint symptoms is an important way to prevent their evolution and deterioration.

Working in the clinic reveals that as people age, most people have knee discomfort, which may be very symptomatic or very mild. The theory of Unpleasant Symptoms was first proposed by American nursing scholars Lenz et al. in 1995 [6], including symptoms, influencing factors, and performance outcomes. In his theory of discomfort symptoms, Lenz emphasized that symptoms are multidimensional and that multiple symptoms coexist and are interrelated. Therefore, based on the Theory of Unpleasant Symptoms [6], we collectively referred to abnormal knee symptoms as knee discomfort. The symptoms include joint pain, numbness, coldness, slight stiffness, slight difficulty in flexion and extension, and increased pain when the temperature drops suddenly [7]. When the symptoms gradually develop into KOA, they seriously lower the quality of life of patients [2]. Currently, there is no cure or spontaneous remission of KOA, and the focus of treatment is symptom relief. People can also gain confidence in managing their KOA with self-management strategies [8]. Therefore, early prevention and self-management of knee health in middle-aged and elderly individuals is particularly important at the early stage of knee discomfort.

Self-management is the process by which individuals with health problems purposefully cooperate with healthcare professionals to perform a series of planned activities [9]. Self-management is a key component of improving care for chronic conditions, with a focus on supporting patients with behavioral skills to manage their condition [10]. Previous studies have examined the relationship between knee symptoms and self-management behaviors. In Ginnerup-Nielsen’s [11] study, 9086 citizens between 60 and 69 years old in the municipality of Frederiksberg, Copenhagen, Denmark, were surveyed. However, this study only examined the relationship between the prevalence of knee pain and self-management behaviors, but not the impact of pain severity and other knee symptoms on self-management behaviors. Other studies have mostly addressed joint symptoms of knee osteoarthritis, preoperative and postoperative knee replacement pain, and knee pain [12], and disease management strategies and clinical practice guidelines remain focused on a single disease. Multiple symptomatic factors of knee discomfort were not considered together, and the survey respondents were all patients with clinically diagnosed knee osteoarthritis or knee arthritis. There was no identification or management of risk factors for patients with early knee arthritis. In terms of survey instruments used, the Chronic Disease Self-Management Behavior Scale developed by Lorig et al [13] was mostly used, including 3 dimensions of cognitive symptom management, exercise, and communication with physicians. According to the Theory of Unpleasant Symptoms, symptoms are influenced by three dimensions: physical, psychological, and environmental, while the current scales lack the investigation of patients’ emotional management and there is no specific scale for patients with knee discomfort. Overall, research on self-management behaviors for knee symptoms is limited. There are indications that sociodemographic factors (e.g., ethnicity/race, gender, age, education) are associated with the decision to use self-management strategies [14]. It is still unclear which demographic characteristics of patients with knee discomfort affect their self-management behaviors.

Our team developed the Knee Discomfort Self-management Behavior Self-rating Scale for middle-aged and elderly people based on symptomatology [7]. The scale is not only applicable to patients with knee osteoarthritis but can also investigate the self-management behavior of patients during periods of knee discomfort without diagnosed knee osteoarthritis, providing a clinical screening for early intervention in patients with KOA. Based on this, we conducted a large sample cross-sectional study to identify the current status and factors influencing self-management in middle-aged and elderly people with knee discomfort, to provide a basis for knee health management.

## Materials and methods

### Study design

This is a large sample cross-sectional study to identify the self-management status of middle-aged and elderly people with knee discomfort and its influencing factors in Hunan Province. We used the STROBE reporting checklist to guide the study reporting.

### Sample size calculation and sampling methods

This study was conducted from January 15 to May 31, 2020. The sample size was calculated using the formula for cross-sectional studies: n = u^2^_α/2_p(1 − p)/d^2^, where u_α/2_=1.96, when α = 0.05, *p* is the prevalence of knee discomfort calculated from pre-experiment (which is 77%), and d is the admissible error (which is 1.2% of *p* in this study). Accounting for a 20% attrition rate, the final required sample size is 9562.

**Fig. 1:**
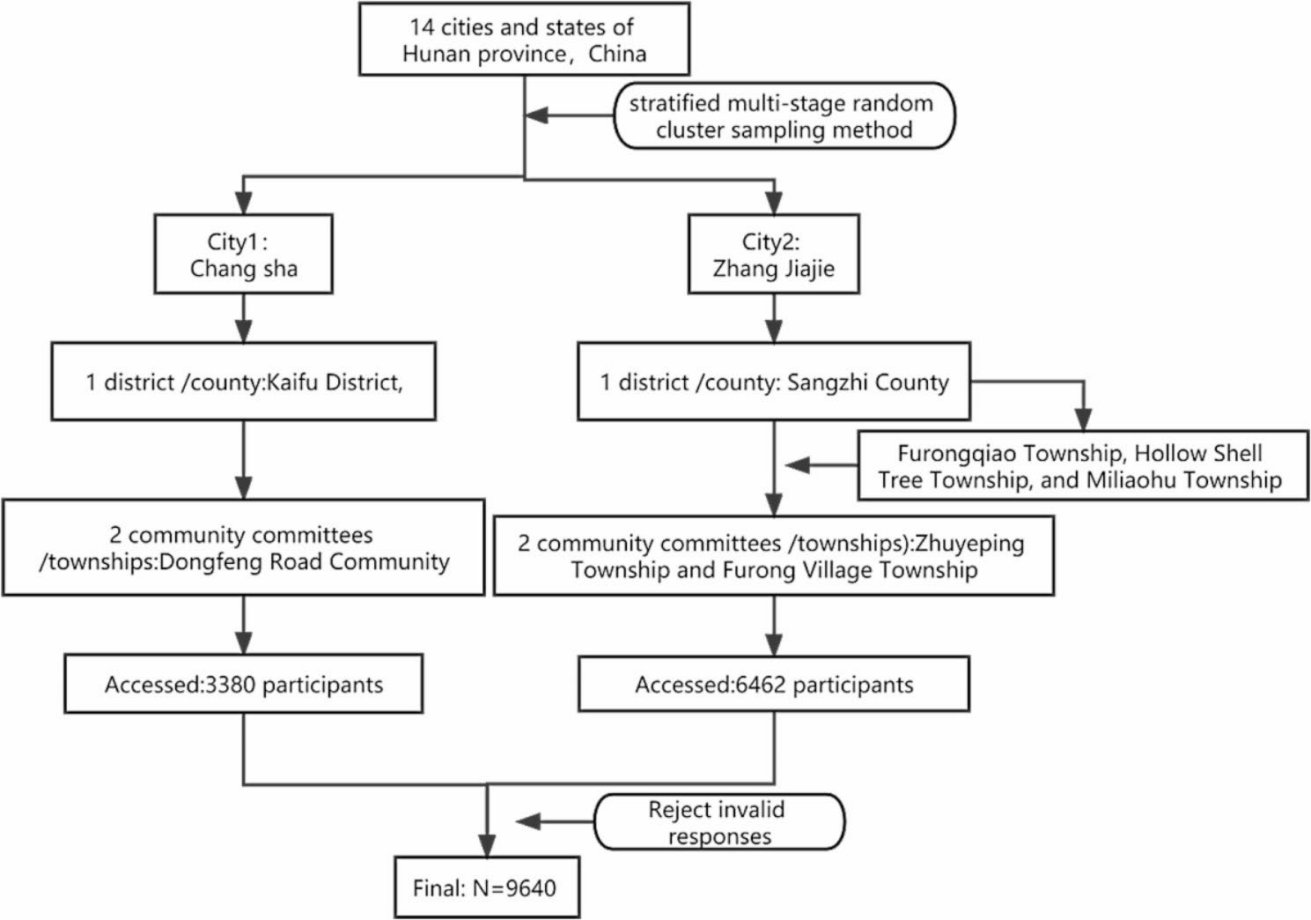
Flow chart for screening the subjects in this study, China, 2020 (N = 9640)

Participants were recruited from Chang Sha and Zhang Jiajie of Hunan Province, China. A stratified multistage random cluster sampling method was used to collect the sample. A total of 10,000 individuals were invited to participate in this study, and 9993 questionnaires were returned. In addition, 353 were ultimately excluded from the analysis due to missing or incomplete data and logical errors(e.g. our scale included reverse-score items, but patients responded to these items in the same way as the others). In total, 9640 valid questionnaires were collected. Figure 1 shows the flow chart for screening the participants. The Ethics Committee of Xiangya Hospital, Central South University approved this study(NO: 202,001,018). All patients are informed and have given their consent. Informed consent was obtained from the legal guardian(s) of the participants which were uneducated.

### Assessment of the knee

People reported knee discomfort based on the initial triage question: “Have you experienced any discomfort from your knee/knees during the last month (both at work and rest)?” Participants who answered “yes” were asked to describe the severity of their discomfort using a 10-point numerical rating scale. “0” was equivalent to no discomfort and “10” reflected the most severe discomfort. If a subject answered “no” to the first question, the score was recorded as 0. Participants who reported discomfort scores from 1 to 3 were considered to have mild knee discomfort, 4 to 6 moderate knee discomfort, and 7 to 10 severe knee discomfort. In addition, the incidence of four common and representative symptoms related to knee discomfort was also investigated: feeling cold in the knee joint, feeling pain in the knee joint, hearing a sound in the knee joint during movement, and experiencing knee joint instability during movement.

### Inclusion and exclusion criteria

The inclusion criteria for participants were (1) 45 years of age or older; (2)able to speak and communicate in Chinese; and (3) volunteered to participate in this study and signed an informed consent form. Individuals were excluded if they had the following conditions: (1) participants with a history of knee surgery; (2) serious illnesses (such as tumors, severe cardiovascular diseases, Parkinson’s, severe mental disorders); (3) unable to complete the questionnaire midway were excluded.

### Measures

#### Demographic and clinical characteristics

A general information questionnaire was designed based on a literature review and consultation with orthopedic experts, encompassing participants’ demographic information and clinical factors such as age, gender, BMI, ethnicity, education level, marital status, household, source of income, monthly income, residence, smoking, drinking alcohol, chronic disease status(e.g., without disease, diabetes, hypertension, and osteoporosis), history of trauma, recurrent knee pain in the last 1 month, and self-reported knee discomfort levels.

#### Knee joint discomfort self-management scale

We used the Knee Joint Discomfort Self-management Scale, developed by Zhao Peipei [7] in 2019, to measure knee joint discomfort self-management among middle-aged and elderly individuals. This scale consists of 27 items: with a 5-point Likert rating (1 = none, 2 = rarely, 3 = sometimes, 4 = often, 5 = always). Each item was scored on a scale from 1 to 5. There were four dimensions in total: symptom management(10 items); daily management(9 items); emotion management(4 items); and information management(4 items); and total scores ranged from 27 to 135. Items 20 and 21 were reverse-scored. The average item score of each dimension = The sum of all dimensional entries/The number of entries for each dimension. Participants who had an average score of < 3 were considered to have a low level of self-management, those with scores from 3 to 4 were categorized as having an average level, and participants with scores ≥ 4 were considered to have a high level of self-management. The validity and reliability of the scale has been previously validated [7]. The overall content validity of the scale was 0.964(0.914 in this study), indicating good internal consistency. The split-half reliability for the scale was 0.894. Test-retest was performed across 23 distinct participants with a correlation coefficient of 0.876. (Details of the scale are in the supporting material).

### Data collection procedure

To ensure the consistency of data, the research assistants who conducted the data collection attended training sessions. All research assistants were asked to complete a questionnaire for this study at the same time. The results of the assessment were checked for discrepancies and the criteria for data collection were standardized to reach a consensus in case of disagreement [10]. Then this study conducted a small-scale presurvey before officially releasing the questionnaire to examine its logic, flow, and comprehensibility. By gathering feedback from participants, it was possible to make timely corrections and improvements to enhance the experience of participants when filling out the questionnaire.

Our research team included 4 community nurses in Chang Sha and Zhang Jiajie cities. They acted as ‘site coordinators’ for the project team and assisted with the recruitment of participants. Community nurses identified potential participants and asked their permission to have the researcher contacti them. If agreeable, the researcher then contacted them and explained the research aim, risks and benefits of participation, and the right to withdraw from the study before data analysis. Subsequently, questionnaires were handed over to participants. Participants who could correctly use electronic products were given electronic questionnaires. Participants who could not use electronic products for reasons, such as advanced age, received paper questionnaires. However, some participants were unable to see or understand the items due to old age or low education levels. In this case, the researchers read the items to them, asked for the participants’ answers, and then filled in the items. Researchers could clarify questions that participants did not understand in ways that did not reveal answers to the participants. Each questionnaire took approximately 15 to 20 min. Participation was voluntary and enthusiastic. All participants were asked to return questionnaires to the researcher in a sealed envelope. After completing the investigation, research assistants reviewed the findings, promptly filled in any missing information, corrected errors, and confirmed the validity of the data.

### Data analysis

Demographic and clinical characteristics for participants were presented as mean (± SD) for continuous variables or percentage (%) for categorical variables. After the Shapiro-Wilk normality test(P < 0.001), the average scores for self-management were not normally distributed. Therefore, the non-parametric test, such as Mann-Whitney U-test and Kruskal-Wallis Test were performed to identify differences in self-management scores according to demographic and clinical characteristics. The Mann-Whitney U-test was used for comparisons of two samples, and the Kruskal-Wallis Test was used for comparisons of two and more samples. Multi-collinearity was checked by variance infation factor (VIF) cut of point < 10. Since the dependent variable “average item score for self-management” was non-normal distribution, a generalized linear regression model was used to analyze the self-management behaviors of middle-aged and elderly people with knee discomfort. Using the average item score for self-management as the dependent variable, only those demographic and clinical factors that were found to be statistically significant in the univariate analysis were included in the generalized linear regression model. The probability distribution is Gamma and the correlation function is “logarithmic”. A two-sided P < 0.05 was considered statistically significant. All statistical analyses were performed using Statistical Package for Social Sciences (SPSS) version 25.0.

## Results

### Participants

A total of 9640 participants completed the questionnaires. The effective recovery rate of the samples was 96.4%. The mean (SD) age of the 9640 participants was 58.99 (12.0), ranging from 45 to 99 years; middle-aged people accounted for 53.2%, and elderly people accounted for 46.8%. The proportions of male(47.3%) and female(52.7%) participants were approximately the same.

### Self-reported knee discomfort degree

**Fig. 2:**
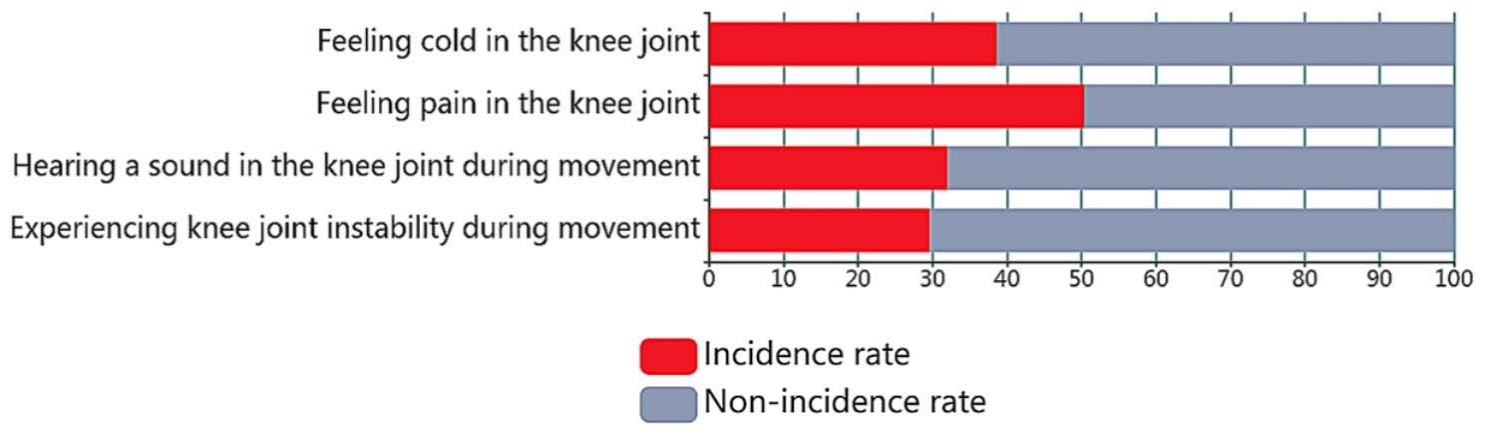
Incidence of common knee joint discomfort symptoms(N = 9640)

Approximately 77% of the elderly people we contacted had self-reported symptoms of knee discomfort. Mild, moderate, and severe knee discomfort accounted for 69.5%, 25.1%, and 5.4%, respectively(in Table 1). Figure 2 shows the incidence of four common and representative symptoms of knee discomfort, namely, feeling cold in the knee joint(39.98%); feeling pain in the knee joint(52.10%); hearing sound in the knee joint during movement(33.16%); and experiencing knee joint instability during movement(30.66%).

**Table 1:** Self-management of the participants with different degrees of knee discomfort(N = 9640)

Variables	Frequency(%)	Management level(case;%)			*X* ^*2*^	*P Value*
		Low	Middle	High		
Mild	6687(69.5)	6159(92.1)	469(7.0)	59(0.9)	176.171	< 0.001
Moderate	2427(25.1)	2056(84.7)	347(14.3)	24(1.0)		
Severe	526(5.4)	418(79.5)	93(17.7)	15(2.9)		

### Scores for self-management and items

Regarding self-management (Table 2), the mean total score for self-management of knee discomfort in middle-aged and elderly individuals was (53.35 ± 20.58). The highest mean total entry score for daily life management was (2.06 ± 0.90), the second-highest mean entry score for emotion management was (1.99 ± 0.67), and the lowest mean entry score for information management was (1.83 ± 0.92). The item with the highest score was “I will have a proper rest (avoid or limit the activity) after the discomfort of the knee joint”(2.37 ± 1.28), and item with the lowestscore was “I will wear knee pads to relieve the symptoms of knee instability” (1.52 ± 0.94).The average score for each item is shown in Table 3.

**Table 2:** Self-management behaviors and total and mean entry scores for each dimension

	Item	Total points	Average entry score (Mean ± SD)	Management level*(case; %)		
Project				Low	Middle	High
Symptom management	10	19.60 ± 8.08	1.96 ± 0.81	8425(87.4)	1057(11.0)	158(1.6)
Daily life management	9	18.46 ± 8.06	2.06 ± 0.90	7861(81.5)	1509(15.7)	270(2.8)
Information management	4	7.32 ± 3.68	1.83 ± 0.92	8043(83.4)	1247(12.9)	350(3.6)
Emotion management	4	7.97 ± 3.71	1.99 ± 0.93	7709 (80.0)	1602(16.6)	329(3.4)
Self-management	27	53.35 ± 20.58	1.98 ± 0.76	8633(89.6)	909(9.4)	98(1.0)

Note: Because of the different number of entries in each dimension, the mean total score and mean entry score were used for the analysis, mean total score = sum of all entries/27; mean entry score of each dimension = sum of entries in each dimension/number of entries in each dimension, mean entry score < 3 was considered a low level of self-management, 3–4 was considered a medium level of self-management, and > 4 was considered a high level of self-management

**Table 3:** Self-management Average Scores of items in the scale

Catalogue	Item	Mean ± SD
**Symptom management**		1.96 + 0.81
	1. I want to relieve my knee joint of unpleasant symptoms	2.23 ± 1.31
	2. I will have a proper rest (avoid or limit the activity) after the discomfort of knee joint	2.37 ± 1.28
	3. If the knee joint is discomfort, I will massage and pat the discomfort part of the knee joint	2.26 ± 1.23
	4. I will raise the knee joint properly after the discomfort	1.97 ± 1.12
	5. After the discomfort of knee joint, I will relieve the symptoms by external application or spreads wipes the medicinal ointment	2.13 ± 1.24
	6. After knee joint discomfort, I will take the medicine according to the doctor’s instructions on time and in quantity	2.06 ± 1.26
	7. I will relieve the symptoms through heat therapy (such as foot soaking, hot compress, fumigation and washing therapy) or cold therapy (such as cold compress, ice massage, etc.)	1.94 ± 1.13
	8. I can relieve symptoms through acupuncture, moxibustion or cupping	1.60 ± 0.95
	9. When walking, I will use crutches, walkers and other auxiliary equipment to relieve symptoms	1.52 ± 0.97
	10. I will wear knee pads to relieve the symptoms of knee instability	1.52 ± 0.94
**Daily life management**		2.06 + 0.90
	11. I will exercise properly according to my physical condition (such as walking slowly, Tai Chi, yoga, swimming, etc.)	1.92 ± 1.16
	12. I will carry out targeted knee exercises according to the doctor’s advice	1.78 ± 1.09
	13. I will pay attention to my own way of eating (such as calcium rich diet, soup for removing dampness, etc.)	2.09 ± 1.20
	14. In order to maintain the knee joint, I will take supplements (such as calcium tablets, vitamins, glucosamine, etc.)	1.97 ± 1.16
	15. I will control my weight when necessary	1.94 ± 1.17
	16. I will reduce other knee bending or weight-bearing actions (such as reducing up and down stairs, climbing, standing for a long time, squatting and kneeling position, etc.)	2.10 ± 1.21
	17. I will change the way I wear shoes (such as comfortable shoes, sports shoes, soft soled shoes, shock absorbing insoles, special shoes and insoles, etc.)	2.15 ± 1.28
	18. I will pay attention to keeping my knees warm (such as adding pants, etc.)	2.36 ± 1.34
	19. I will take the initiative to improve the living environment (such as keeping the environment dry and warm, avoiding living in dark and humid places, etc.)	2.14 ± 1.34
**Emotion management**		1.99 + 0.93
	20. The discomfort of knee joint has always existed, and I can’t adapt to it	1.79 ± 1.10
	21. I will worry about the aggravation of discomfort of knee joint, which will affect my life	1.96 ± 1.20
	22. I can solve the discomfort and trouble caused by the discomfort of knee joint by myself, and be optimistic	2.23 ± 1.33
	23. When the knee joint is discomfort, I will divert my attention by doing other things (such as watching TV, playing mahjong, talking, seeking comfort, etc.)	1.98 ± 1.19
**Information management**		1.83 + 0.92
	24. After knee joint discomfort, I will ask for help from people with medical professional background	1.97 ± 1.17
	25. I will talk with other residents with knee joint disease about the knowledge of the disease	1.85 ± 1.11
	26. I get information about my illness by Internet (such as online query, wechat push, watching TV, etc.)	1.79 ± 1.09
	27. I get information about my illness by consulting medical books, newspapers and magazines etc.	1.71 ± 1.04

### Factors influencing self-management in middle-aged and elderly individuals

Table 4 summarizes a comparison of knee joint discomfort self-management scores among participants by demographic and clinical characteristics. Self-management of knee discomfort was associated with age, gender, ethnicity, education level, marital status, source of income, income per month, long-term residence status, presence of chronic disease(e.g., without disease, diabetes, hypertension, and osteoporosis), history of trauma, recurrent knee pain in the past month and self-reported knee discomfort (p<0.05).

**Table 4:** Comparison of knee joint discomfort self-management scores among participants with demographic and clinical characteristics (N = 9640)

Variables	Categories	Frequency	Mean(SD)	*H or Z*	*P Value*
*Socioeconomic demographic characteristics*					
Age(Years)	<60	5192	1.94(0.76)	-5.213	< 0.001*
	≥ 60	4448	2.02(0.76)		
Gender	Male	4559	1.92(0.76)	-7.365	< 0.001*
	Female	5081	2.03(0.76)		
BMI	<18.5	1380	1.95(0.76)	4.755	0.093
	18.5–24	5893	1.98(0.76)		
	>24	2367	1.95(0.76)		
Ethnicity	Ethnic Han	4979	2.00(0.77)	-3.179	0.001*
	Ethnic minorities	4661	1.95(0.75)		
Educational level	Primary school and less	7031	1.98(0.75)	28.009	< 0.001*
	Middle school	1216	1.92(0.76)		
	University and more	1393	1.98(0.79)		
Marital status	Unmarried	348	1.85(0.80)	30.669	< 0.001*
	Married	8355	1.97(0.76)		
	Divorced	211	2.02(0.74)		
	Widowed	726	2.08(0.75)		
Household	Living alone	773	1.97(0.78)	3.823	0.148
	Living with family	8442	1.98(0.76)		
	Nursing home or elsewhere	425	1.91(0.76)		
Source of income	Occupational wages, farming	5582	1.95(0.76)	-4.582	< 0.001*
	Pension, spouse, children, other	4058	2.02(0.77)		
Income per month(RMB)	< 1000	2248	1.97(0.75)	28.346	< 0.001*
	1000–3000	4041	1.93(0.74)		
	3001–5000	2036	2.01(0.77)		
	5001–10,000	945	2.05(0.81)		
	> 10,000	370	2.06(0.79)		
Long-term residence	City	2915	2.02(0.79)	-3.117	0.002*
	country	6725	1.96(0.75)		
Smoke	No	7413	1.98(0.76)	-0.889	0.374
	Yes	2227	1.97(0.77)		
Drinking alcohol	No	6187	1.97(0.76)	3.558	0.169
	Light alcohol on occasion	3171	1.99(0.76)		
	Alcoholic	282	1.96(0.80)		
***Clinical characteristics***					
Do you have a chronic disease	No	3369	1.78(0.76)	-20.291	< 0.001*
	Yes	6271	2.08(0.74)		
Diabetes	No	8970	1.96(0.76)	-6.483	< 0.001*
	Yes	670	2.15(0.76)		
Hypertension	No	7601	1.92(0.76)	-13.932	< 0.001*
	Yes	2039	2.18(0.75)		
Osteoporosis	No	8373	1.93(0.75)	-16.960	< 0.001*
	Yes	1267	2.31(0.74)		
History of trauma	No	8035	1.96(0.76)	-6.124	< 0.001*
	Yes	1605	2.08(0.75)		
Recurrent Knee pain in the past month	No	7146	1.84(0.74)	-30.781	< 0.001*
	Yes	2494	2.36(0.68)		
Self-report knee discomfort	Mild	6687	1.83(0.75)	871.358	0.001*
	Moderate	2427	2.28(0.68)		
	Severe	526	2.38(0.75)		

**Note: SD**, Standard Deviation; **H**, statistic of Kruskal-Wallis Test; **Z**, statistic of Mann-Whitney U-test; * signifcant association(*P* Value < 0.05)

Self-management scores were higher in participants aged over 60 than those under aged 60(Z=-5.213,p < 0.001); female patients had higher scores than males (Z =-7.365, p< 0.001); patients with Han nationality had higher scores than those with minority ethnicity(Z=-3.179, p < 0.001); patients with middle school education or more had better self-management than those with primary school education or less(H = 28.009, p < 0.001) There were differences in the scores of people with different marital statuses(H = 30.669, p < 0.001; participants with income from children had higher self-management scores than those who worked or farmed(Z=-4.582, p < 0.001); and patients with a family income of more than 10,000 RMB compared to those with lower family income, had a higher self-management scores(H = 28.346, p < 0.001). People who had lived in cities for a long time had higher scores for self-management than those who lived in rural areas(Z=-3.117, p = 0.002). People with a history of chronic disease(such as diabetes, hypertension, and osteoporosis), trauma, and self-reported knee pain within a month scored higher than those without(Z=-20.291, p < 0.001; Z=-6.124, p < 0.001; Z=-30.781, p < 0.001). There was no difference in scores between people with different BMIs(H = 4.755, p = 0.093).

A generalized linear regression analysis was performed with the mean entry score of self-management behavior as the dependent variable and 13 variables with statistical significance in the univariate analysis as independent variables. Finally, gender**(***β = 0.065*, Wald*X*^*2*^ = 19.921, p < 0.001), ethnicity (*β=* -0.06, Wald*X*^*2*^ = 15.468, p < 0.001), and education level(*β*_Middle school_ *=* 0.056, Wald*X*^*2*^ = 5.888, p = 0.015; *β*_University and above_ *=* 0.163, Wald*X*^*2*^ = 36.743, p < 0.001), source of income(*β =* 0.033, Wald*X*^*2*^ = 4.649, p = 0.031), monthly income(*β*_3001-5000_ *=* 0.088, Wald*X*^*2*^ = 14.712, p < 0.001; *β*_5001-10000_ *=* 0.113, Wald*X*^*2*^ = 13.408, p < 0.001; *β*_10000_ *=* 0.091, Wald*X*^*2*^ = 4.408, p = 0.036 ), without disease(*β=*-0.112, Wald*X*^*2*^ = 43.277, p < 0.001), hypertension(*β =* 0.081, Wald*X*^*2*^ = 17.898, p < 0.01), osteoporosis(*β =* 0.174, Wald*X*^*2*^ = 61.355, p < 0.001), recurrent knee pain in the past 1 month(*β = 0.36*, Wald*X*^*2*^ = 402.011, p < 0.001), and self-reported discomfort (*β*_Moderate_ *=* 0.318, Wald*X*^*2*^ = 309.62, p < 0.001; *β*_Severe_ *=* 0.371, Wald*X*^*2*^ = 121.663, p < 0.001) were identified as factors influencing self-management in the middle-aged and elderly participants. Details are shown in Table 5 and Supporting Material 2.

**Table 5:** Generalized linear regression analysis of factors influencing self-management behavior

Variable	*β*	SE	95% *CI*	Wald*X*^*2*^	*P* Value
**Female**	0.065	0.015	0.036–0.093	19.921	< 0.001*
**Ethnic minorities**	-0.06	0.015	-0.089-(-0.03)	15.468	< 0.001*
**Educational level**					
Middle school	0.056	0.023	0.011–0.101	5.888	0.015*
University and more	0.163	0.027	0.11–0.215	36.743	< 0.001*
**Source of income**					
Pension, spouses, children, others	0.033	0.016	0.003–0.064	4.649	0.031*
**Income per monthly(RMB)**					
3001–5000	0.088	0.023	0.043–0.134	14.712	< 0.001*
5001–10,000	0.113	0.031	0.052–0.173	13.408	< 0.001*
>10,000	0.091	0.044	0.006–0.176	4.408	0.036*
**Without chronic disease**	-0.112	0.017	-0.146-(-0.079)	43.277	< 0.001*
**Hypertension**	0.081	0.019	0.044–0.119	17.898	< 0.001*
**Osteoporosis**	0.174	0.022	0.131–0.218	61.355	< 0.001*
**Recurrent knee pain in the past month**	0.36	0.018	0.325–0.396	402.011	0.001*
**Self-report knee discomfort**					
Moderate	0.318	0.018	0.282–0.353	309.62	< 0.001*
Severe	0.371	0.034	0.305–0.437	121.663	< 0.001*

**Note: SE**, standard error; * signifcant association(*P* Value < 0.05)

## Discussion

Currently, in China, over 50% of chronic disease patients aged 65 and older suffer from osteoarthritis. This condition is an inconvenience, restricts their activities and leads to a decrease in their quality of life. [15]. Guidelines for the treatment of knee osteoarthritis indicate that the ability to self-manage is a good way for patients to improve their condition [5]. To our knowledge, this is the first cross-sectional study based on patient self-reported discomfort symptoms to identify factors influencing self-management among middle-aged and elderly people with knee discomfort, so that interventions could be applied to prevent symptoms from worsening.

Patient-reported outcome refers to the subjective evaluations of patients regarding their symptoms, functional status, and quality of life [16]. Symptom management is the basis of clinical medical care, and timely and effective symptom management is very important to improve patient clinical outcomes and satisfaction [17]. Approximately 77% of the elderly people we contacted had self-reported symptoms of knee discomfort. The most common symptom of knee joint discomfort was feeling pain in the knee joint(52.10%). We found that knee discomfort self-management among these participants was at a low level, suggesting that self-management of knee discomfort in this population needs to be further improved. However, it is difficult to directly compare our results with prior studies because of the lack of studies using the same instrument as the current study. By analyzing various dimensions, it was discovered that participants performed the worst in the information management dimension, indicating that they have difficulty accessing medical information to help relieve their symptoms. Currently, most information can be accessed from the internet, but some elderly individuals have difficulty mastering the internet, which affects their access to information to a certain extent [18]. It may be difficult for them to manage themselves when faced with complex situations such as how to manage symptoms and how to effectively control knee discomfort in daily life. This may be because the patients themselves do not attach enough importance to discomfort and choose to ignore it [4] when it does not affect their daily life or when the symptoms are mild and are not managed in time. It is also worth noting that middle-aged and elderly people are in the emotional management dimension of knee discomfort management, perhaps since middle-aged and elderly people are not willing to talk about their emotions, resulting in a poor emotional state [19], or they are unable to recognize their emotions correctly and have difficulties managing the negative emotions caused by knee discomfort. Therefore, healthcare professionals should prioritize improving the self-management of middle-aged and elderly individuals in terms of information management, symptom management, daily life management, and emotional management. Particularly, emphasis should be placed on information management. Healthcare professionals can engage in informal discussions with patients to understand their concerns regarding medical conditions, thereby developing information frameworks to enhance awareness of clinical characteristics and misconceptions related to knee joint diseases. Furthermore, mobile-based information support has been shown to have application value in enhancing patient self-management [20]. In the future, mobile devices can serve as a platform to provide automated and personalized short messages based on information frameworks directed at middle-aged and elderly individuals with knee joint discomfort to support their daily life, symptoms, and emotional management. This approach is both practical and cost-effective for primary healthcare institutions.

Our study identified socioeconomic characteristics associated with self-management of knee discomfort in middle-aged and elderly adults, which included gender, ethnic minorities, education level, source of income, and monthly income. Similar to previous findings, women had better self-management skills than men, and they were more likely to seek social interaction resources, such as educational courses and support groups [21]. At the national level, China is composed of 56 ethnic groups, except for the Han, the rest are ethnic minorities. In this study, the self-management ability of the elderly individuals who were members of an ethnic minority was lower than those who were Han, suggesting that we should pay attention to the health of this population. Generally, consistent with previous findings, the study found that patients with higher education levels were more likely to actively seek disease-related knowledge and resources and discuss their condition and treatment plans with healthcare professionals. This is consistent with a previous study on people with type 2 diabetes [22]. Understanding the economic factors of patients can provide tailored support and resources for individuals facing financial barriers. The association between income(e.g. source of income, monthly income) and self-management observed in this study is consistent with the findings of previous studies, in which economic factors were associated with self-management in adults with Parkinson’s disease [10]. In our study, participants who were financially supported by children’s or retirement wages and who had higher monthly income had better self-management. This suggests that support from family is an enabling factor for self-management in patients with knee discomfort. The family plays a key role in building a positive environment and plays an important role in supporting and promoting self-management of chronic diseases. Family support provides both adequate material resources for patients’ self-management and indirectly reflects family members’ expectations of good behavior in patients, which in turn leads to more positive perceptions of self-management behaviors, such as dietary modification, and physical exercise [23]. Therefore, healthcare professionals should also assess the level of family support for patients with knee joint discomfort. Developing and implementing family-based self-management intervention plans may greatly improve the management behaviors of middle-aged and elderly individuals with knee joint discomfort, thereby effectively preventing the development of knee arthritis.

In addition to socioeconomic demographic factors, this study considered clinical characteristics that influence knee discomfort self-management, including the presence of chronic disease, recurrent knee pain in the past 1 month, and self-reported discomfort as significant factors. Kang et al. [24] pointed out that the self-management of patients with multiple chronic diseases is relatively complex, resulting in poor self-management behavior. However, in our study, different results emerged. Chronic disease participants had better knee discomfort self-management than those without chronic diseases. It has been shown that participation in the care of patients with chronic diseases improves self-management and problem-solving skills [25]. Therefore patients are also motivated to adopt healthier lifestyles when managing their comorbidities. In our study, the most common coexisting chronic disease among the elderly participants was hypertension. However, it was found that compared to hypertension, osteoporosis had a greater impact on the self-management of knee joint discomfort(β = 0.174, Wald X2 = 61.355, p < 0.001). This may be because both osteoporosis and knee joint discomfort are manifestations of musculoskeletal symptoms. Participants with recurrent knee pain in the past 1 month had relatively stronger self-management abilities. It may be that previous pain makes the patient more aware of the management of the knee and prevents the recurrence of uncomfortable symptoms [26]. The results of self-reported knee discomfort were similar to those of a previous study, which showed that clinical pain severity is closely related to self-management behaviors [27]. Therefore, healthcare professionals should consider assessing clinical characteristics such as chronic diseases and recent knee joint pain within the past month in patients with knee joint discomfort.

This study also showed that the level of self-reported knee discomfort affected self-management(p < 0.001). The more severe the knee discomfort, the better the self-management behavior. Patients selectively ignore mild knee discomfort, while severe symptoms require more self-management to relieve and control. This may be related to a lack of awareness or perception of risk among the patients. As in a meta-analysis [28] of self-management behaviors in women with diabetes during pregnancy, low-risk perception was shown to be a barrier to lifestyle change in patients. How to effectively enhance patients’ risk awareness and improve the management of knee discomfort should be a key focus of future research. For instance, developing smartphone applications or knee joint health monitoring devices can offer personalized advice and reminders, making it convenient for patients to track and manage their knee joint issues.

Therefore, in terms of self-management strategies for patients with knee discomfort, tailor-made, ongoing support of healthcare professionals, and the encouragement of family and friends are required to assist patients [29, 30]. It will enhance patients’ motivation to self-manage their behavior [31], motivating them to seek additional health information resources from health care providers. Additionally, it is important to raise risk perception awareness among middle-aged and elderly individuals, to strengthen their understanding of the significance of knee discomfort and prevent the worsening of symptoms.

### Research limitations

The strengths of this study are the large sample size and the scale used in this study was developed specifically to measure the self-management of patients with knee discomfort. The four dimensions of the scale can also be used as a theoretical basis for the development and observation of the effects of self-management programs for patients with knee discomfort in the future. However, some limitations are as follows. First, the study was performed in the Hunan province of China, so the research is explorative, which might influence the generalizability of the results. The reliability and validity of this Knee Joint Discomfort Self-management Scale have been established only in Chinese participants. Additionally, this study only analyzed the influence of sociodemographic data on the self-management behavior of the study participants. Finally, there are few previous studies investigating knee self-management based on symptoms, so it is difficult to compare this study with previous studies.

## Conclusions

In conclusion, knee discomfort self-management behaviors of middle-aged and elderly people are at a low level. Healthcare providers assessing self-management in middle-aged and elderly individuals should consider socioeconomic demographic factors. Additionally, attention should be given to improving the ability of middle-aged and elderly individuals and their family members to access health information and encouraging them to actively seek health care resources and social support, such as family members. In this study, the more severe the knee discomfort was, the better the self-management behavior, indicating that attention should also be given to increasing individuals’ awareness of disease risks to promote self-management. Finally, it is suggested to develop personalized interventions from the perspective of symptom management, daily life management, information management, and emotional management as well as the factors influencing self-management to improve individuals’ self-management abilities.

### Electronic supplementary material

Below is the link to the electronic supplementary material.

Supplementary Material 1

## Data Availability

The datasets of this study are available from the corresponding author on reasonable request.
