# Supplementary material for: Current status and influencing factors of self-management in knee joint discomfort among middle-aged and elderly people: a cross-sectional study
Source: BMC Geriatr. 2023 Sep 29;23:612. doi: 10.1186/s12877-023-04334-x (PMC10541685; doi:10.1186/s12877-023-04334-x)
Supplement: Supplementary file 1 — Supplementary Material 1 [file 12877_2023_4334_MOESM1_ESM.docx]

**Supporting material1 Knee joint discomfort Self-management Scale**

| ITEM |
| --- |
| 1. I want to relieve my knee joint of unpleasant symptoms |
| 2. I will have a proper rest (avoid or limit the activity) after the discomfort of knee joint |
| 3. If the knee joint is discomfort, I will massage and pat the discomfort part of the knee joint |
| 4. I will raise the knee joint properly after the discomfort |
| 5. After the discomfort of knee joint, I will relieve the symptoms by external application or spreads wipes the medicinal ointment |
| 6. After knee joint discomfort, I will take the medicine according to the doctor's instructions on time and in quantity |
| 7. I will relieve the symptoms through heat therapy (such as foot soaking, hot compress, fumigation and washing therapy) or cold therapy (such as cold compress, ice massage, etc.) |
| 8. I can relieve symptoms through acupuncture, moxibustion or cupping |
| 9. When walking, I will use crutches, walkers and other auxiliary equipment to relieve symptoms |
| 10. I will wear knee pads to relieve the symptoms of knee instability |
| 11. I will exercise properly according to my physical condition (such as walking slowly, Tai Chi, yoga, swimming, etc.) |
| 12. I will carry out targeted knee exercises according to the doctor's advice |
| 13. I will pay attention to my own way of eating (such as calcium rich diet, soup for removing dampness, etc.) |
| 14. In order to maintain the knee joint, I will take supplements (such as calcium tablets, vitamins, glucosamine, etc.) |
| 15. I will control my weight when necessary |
| 16. I will reduce other knee bending or weight-bearing actions (such as reducing up and down stairs, climbing, standing for a long time, squatting and kneeling position, etc.) |
| 17. I will change the way I wear shoes (such as comfortable shoes, sports shoes, soft soled shoes, shock absorbing insoles, special shoes and insoles, etc.) |
| 18. I will pay attention to keeping my knees warm (such as adding pants, etc.) |
| 19. I will take the initiative to improve the living environment (such as keeping the environment dry and warm, avoiding living in dark and humid places, etc.) |
| 20. The discomfort of knee joint has always existed, and I can't adapt to it |
| 21. I will worry about the aggravation of discomfort of knee joint, which will affect my life |
| 22. I can solve the discomfort and trouble caused by the discomfort of knee joint by myself, and be optimistic |
| 23. When the knee joint is discomfort, I will divert my attention by doing other things (such as watching TV, playing mahjong, talking, seeking comfort, etc.) |
| 24. After knee joint discomfort, I will ask for help from people with medical professional background |
| 25. I will talk with other residents with knee joint disease about the knowledge of the disease |
| 26. I get information about my illness by Internet (such as online query, wechat push, watching TV, etc.) |
| 27. I get information about my illness by consulting medical books, newspapers and magazines etc, |

**Supporting material 2 Independent variable assignment method**

| Variable | **Assignment Methods** |
| --- | --- |
| Gender | Male=0; Female =1 |
| Ethnicity | Ethnic Han=1, Ethnic minorities=2 |
| Education level | Primary school and less=1；Middle school=2；University and more=3 |
| Source of income | Professional wages, Farming=1; Retirement benefits, spouses, children., others=2 |
| Income per monthly | <1000=1; 1000-3000=2; 3001-5000=3; 5001-10000=4; >10000=5 |
| without chronic disease | No=0; Yes=1 |
| Hypertension | No=0; Yes=1 |
| Osteoporosis | No=0; Yes=1 |
| Recurrent knee pain in the past month | No=0; Yes=1 |
| Self-reported knee discomfort | Without discomfort=0; Mild=1; Moderate=2; Severe=3 |
| Y | Average item score of self-management |
